# Extrapyramidal Syndrome due to Aripiprazole Overdose in a Young Woman: An Unusual Case Report

**DOI:** 10.1155/2024/8883047

**Published:** 2024-08-05

**Authors:** Homa Talabaki, Ensiyeh Taghizadeh, Zakaria Zakariaei

**Affiliations:** ^1^ Department of Clinical Pharmacy Faculty of Pharmacy Mazandaran University of Medical Sciences, Sari, Iran; ^2^ Department of Emergency Medicine Faculty of Medicine Mazandaran University of Medical Sciences, Sari, Iran; ^3^ Toxicology and Forensic Medicine Division Mazandaran Registry Center for Opioids Poisoning Antimicrobial Resistance Research Center Imam Khomeini Hospital Mazandaran University of Medical Sciences, Sari, Iran

## Abstract

Aripiprazole is an atypical antipsychotic medication indicated for the treatment of schizophrenia and bipolar disorders. The drug has been shown to exhibit acceptable efficacy and is often preferred as a first-line psychiatric treatment option owing to its lower incidence of adverse effects. While first-generation antipsychotics are associated with extrapyramidal syndrome (EPS), atypical antipsychotics such as aripiprazole are generally associated with a lower frequency of EPS. In this case, we present a 31-year-old woman with a history of bipolar disorder who developed EPS after ingesting 200 mg of aripiprazole. Fortunately, her symptoms improved with the administration of biperiden, and she was discharged five days after ingestion. This case highlights the potential for significant consequences associated with aripiprazole, even within its therapeutic index.

## 1. Introduction

Aripiprazole is an atypical antipsychotic medication indicated for the treatment of schizophrenia and bipolar disorders. The drug has been shown to exhibit acceptable efficacy and is often preferred as a first-line psychiatric treatment option owing to its lower incidence of adverse effects. The side effects associated with aripiprazole include weight gain, elevation of prolactin levels, QTc prolongation, and sedation [1].

In contrast to first-generation antipsychotics (FGAs), aripiprazole acts as a partial agonist at dopamine D2 receptors and as an antagonist at serotonin 5-HT2A receptors. The antagonism of the drug towards 5-HT2A receptors results in heightened dopamine release within the striatum, consequently diminishing the degree of dopamine blockade [2]. Aripiprazole also exerts an effect on histamine-1 receptors and alpha-1 adrenergic receptors. The alpha-1 adrenergic receptor blockade is associated with the occurrence of orthostatic hypotension [3]. Due to its unique pharmacological profile, aripiprazole has a lower likelihood of causing extrapyramidal syndrome (EPS) in comparison to other atypical antipsychotics [4]. Aripiprazole has the potential to induce EPS such as akathisia and parkinsonism, which can be distressing and may lead to treatment discontinuation. When used in adults as an augmentation therapy for depression, aripiprazole can lead to EPS in a dose-dependent manner, with the prevalence of these side effects increasing from 4.7% to 26% as the dosage is escalated [5]. Overdose with aripiprazole is uncommon, and the most commonly observed side effect is sedation [6].

Extrapyramidal syndrome (EPS) is a group of adverse drug reactions that can occur with the use of antipsychotic medications. The EPS includes limb rigidity, tremor, and other manifestations that resemble Parkinson's disease. These reactions are caused by a dysfunction in the extrapyramidal nerve pathway. In addition to parkinsonian-like symptoms, dystonia (abnormal movements of the face and body), akathisia (restlessness or an inability to sit still), and tardive dyskinesia (involuntary repetitive body movements, such as grimacing and sticking out the tongue) are also common EPS. Patients with psychosis and related conditions who are being treated with antipsychotic medications often experience extrapyramidal side effects. These side effects are named as such because they arise from dysfunction in the extrapyramidal framework, which is a neural network in the brain responsible for controlling posture, muscle tone, and coordination [7].

Among the atypical antipsychotics, clozapine has the lowest risk of EPS, while risperidone has the highest. Risk factors for EPS include a history of prior EPS and a high medication dosage. Elderly females are more susceptible to drug-induced parkinsonism and tardive dyskinesia, while young males are more likely to manifest dystonic reactions. It has also been noted by some authors that the pediatric population is more likely to develop EPS while under treatment with (atypical) antipsychotics [8, 9]. In contrast, meta-analysis reveals that the incidence of EPS in children and adolescents treated with aripiprazole is not negligible [10].

In this case report, we present the experience of a woman with a history of bipolar disorder who exhibited EPS after ingesting a 200 mg aripiprazole tablet. The aim of this report is to underscore the relatively lower incidence of EPS linked to atypical antipsychotics when compared to typical antipsychotics. It is important to note that even with low doses of aripiprazole, this complication can arise. This information may assist clinicians in making more informed treatment decisions and in closely monitoring the use of aripiprazole.

## 2. Case Presentation

A 31-year-old woman with a history of bipolar disorder was brought to the emergency room (ER) of a hospital in northern Iran by her family, 12 hours after intentionally overdosing on aripiprazole (200 mg). Six months prior, the psychiatrist had prescribed a dosage of 10 mg tablets of aripiprazole per day (a total of 30 tablets per month) for the patient; upon entering the ER, the patient exhibited coarse hand tremors and muscular dystonia, a condition characterized by the sustained, repeated, or twisted muscle contractions. The coarse tremors were involuntary and moderate rhythmic movements, the severity of which increased upon stimulation and arousal. Muscular dystonia was primarily observed in the neck (cervical dystonia), face, eyelids (blepharospasm), and limbs, resulting in a series of sustained and twisted muscle contractions. She suffered from bipolar disorder for 10 years and had no other underlying disease. The patient had been taking aripiprazole for approximately 6 months prior to presentation, and her medical history did not indicate any previous episodes of EPS while on a therapeutic dose of the medication.

Her initial vital signs were recorded as follows: heart rate of 90 beats per minute, respiration rate of 18 breaths per minute, blood pressure reading of 119/76 mm Hg, temperature of 37°C, and pulse oximetry reading of 98% on room air. In addition to the coarse tremors and muscular dystonia, the patient displayed lethargy, miotic pupils that reacted to light, flushing on the face, dryness of the mouth and mucosa, and warm skin upon examination. The patient's level of consciousness was assessed at 13 out of 15 based on the Glasgow Coma Scale, indicating drowsiness and the ability to open their eyes in response to sound.

Due to the patient's confusion, she exhibited no orientation to person, place, or time and underwent a normal neurological examination, including a normal gait. Laboratory investigations, including electrolyte levels and a complete blood count, were within normal limits. Urinalysis and a urine immunoassay drug screen for benzodiazepines, barbiturates, cannabinoids, amphetamines, cocaine, and opioids were negative. In addition, serum concentrations of ethanol, salicylate, and acetaminophen were nondetectable. A 12-lead electrocardiogram (ECG) was performed, which yielded normal results. Based on these assessments, the patient was subsequently transferred to the toxicology ward for further management.

In order to address the aripiprazole overdose and its associated symptoms, the patient was administered anticholinergic and benzodiazepine medications. Specifically, biperiden ampoule, an anticholinergic agent, was given at a dosage of 5 mg every 12 hours for a total duration of 24 hours. Furthermore, midazolam ampoule, a benzodiazepine, was infused intravenously at a rate of 1 mg/hour for a period of 6 hours. After the completion of the infusion, the patient's EPS symptoms were resolved. Following that, the patient started taking biperiden tablets at a dose of 2 mg every 12 hours for 48 hours.

After a 5-day period, the patient was discharged from the toxicology ward and subsequently transferred to the psychiatric department for further evaluation. Upon discharge from the hospital, the patient was referred to a psychology clinic where she received further treatment from a psychiatrist who prescribed aripiprazole tablets. During the 6-month follow-up, she exhibited a stable psychological condition. This study was conducted according to the Declaration of Helsinki Principles. Also, CARE guidelines and methodology have been followed in this study.

## 3. Discussion

Aripiprazole is primarily metabolized in the liver through two P450 isozymes, CYP2D6 and CYP3A4, and its metabolites are primarily eliminated in the urine and feces. Due to the long elimination half-life of both the parent compound (75 hours) and its active metabolite, dehydroaripiprazole (95 hours), a longer observation period may be necessary after an overdose to monitor for clinical manifestations [11]. Although supratherapeutic ingestions have resulted in persistent symptoms for several days, symptom onset is typically observed within three hours of ingestion, as reported in multiple cases [3, 6]. It is important for healthcare providers to carefully monitor patients who have overdosed on aripiprazole and to provide appropriate medical care and symptomatic treatment as necessary.

While current guidelines generally do not recommend the prophylactic or long-term use of anticholinergics in schizophrenic patients taking antipsychotics, this decision should be made on a case-by-case basis with meticulous risk-benefit analysis [12].

The emergence of EPS among patients suffering from schizophrenia is linked with suboptimal compliance to other atypical antipsychotic medications, which can result in subsequent relapse of the disease and subsequent hospitalization. Inadequate diagnosis and management of EPS have been associated with the manifestation of suicidal thoughts, aggressive behavior, and violent tendencies. Prolonged episodes of dystonia induced by certain medications can lead to the rare but serious complication of rhabdomyolysis [12]. Acute laryngeal dystonia is a dystonic disorder that occurs infrequently but poses a grave risk as it can lead to sudden death resulting from upper airway obstruction [13]. Restoring the equilibrium between acetylcholine and dopamine in the striatum has been shown to decrease EPS. This is why low-potency FGAs with significant anticholinergic activity, such as chlorpromazine and thioridazine, may present fewer challenges in this regard compared to high-potency FGAs. In addition, this balance can be achieved through the use of anticholinergics such as benztropine or trihexyphenidyl, amantadine, dose reduction of the antipsychotic, or by opting for a second-generation antipsychotics instead [14].

Acute dystonic reactions typically manifest soon after the initiation of treatment or following a dose increase and can be effectively managed using intravenous administration of diphenhydramine (25–50 mg) or benztropine (1–2 mg). To ensure a sustained effect of the medication, oral administration of diphenhydramine (25–50 mg) three or four times daily or benztropine (1–2 mg) twice daily should be continued for 2–3 days after the parenteral treatment [15]. Benzodiazepines such as lorazepam (1–2 mg IV in adults; 0.05 mg/kg IV in children), amantadine (100 mg orally twice or thrice daily in adults), and biperiden (2 mg orally in adults) are among the alternative treatments available for EPS [16].

Mazer et al. reported the case of a 14-year-old boy with a history of depression who presented with symptoms of drowsiness, irritability, and acute dystonia in the form of tongue and jaw fasciculation after an overdose of 100 mg of aripiprazole. The patient's dystonia was successfully treated with diphenhydramine [17]. In the case described, the patient experienced EPS after consuming 200 mg of aripiprazole, but fortunately, her symptoms improved with the administration of biperiden.

A recent study revealed that a 27-year-old woman intentionally consumed 330 mg of aripiprazole. In addition, she ingested one 10-mg cyclobenzaprine tablet and one 25-mg quetiapine tablet with suicidal intent. Surprisingly, the patient experienced only mild sedation and no other adverse effects. As a result of these findings, researchers proposed that the therapeutic index of aripiprazole in cases of intentional overdose is significantly broad. Furthermore, serum level assessments conducted by the drug's manufacturer indicated that the total levels of aripiprazole and dehydroaripiprazole reached 716 ng/mL, which is approximately six times higher than the peak concentration associated with a therapeutic dose of 30 mg [18].

In another study, a case of aripiprazole overdose in a child was documented, presenting symptoms of prolonged lethargy, EPS, and ECG changes. It was observed that in overdose cases, the lethargy experienced was self-limiting, and airway patency and respiratory drive were maintained. While lethargy has been consistently reported in instances of aripiprazole overdose in younger children, EPS is only occasionally described. Dystonic reactions from aripiprazole are infrequent. Notably, the patient's symptoms, including tremors and spasticity, had resolved 72 hours after ingesting 52 mg of aripiprazole [19].

A study involving a 3-year-old child who accidentally ingested 200 mg of aripiprazole revealed the development of tongue fasciculations, arm twitching, suppressible rhythmic jaw movements, and ataxia. It was observed that this dose led to immediate lethargy, with a brief improvement noted 16 hours after ingestion, followed by a subsequent decline 2 hours later. However, the patient returned to baseline 72 hours after ingestion [20]. One case reported a six-year-old boy who developed flaccid facial muscles and drooling after the ingestion of aripiprazole 10 mg, which was successfully treated with diphenhydramine 25 mg [21].

In a study involving 239 cases treated with aripiprazole, based on patient medical records and the Danish National Hospital Register, researchers discovered that aripiprazole overdose was generally well tolerated and did not result in short-term fatalities. However, they did not find any correlation between the ingested dose and the presence of symptoms. The most prevalent symptom observed was mild sedation in both the single- and mixed-drug exposure groups. In addition, clinical features associated with EPS, such as tremor and dystonia, were reported in three cases within the single-drug exposure group [22].

### 3.1. Future Priorities

There are several gaps in the available studies on EPS associated with atypical antipsychotics such as aripiprazole. Most of the studies primarily focused on symptomatic outcomes and have not measured functional outcomes. In addition, there is a need for studies that examine the specific mechanisms of atypical antipsychotics that contribute to the development of EPS. Furthermore, there is a need for clinical trials to evaluate the incidence of this phenomenon in patients with different underlying diseases and conditions that require the use of aripiprazole.

## 4. Conclusion

Based on this case, it is possible that aripiprazole toxicity can lead to the occurrence of extrapyramidal syndrome (EPS). Prompt administration of anticholinergic agents such as diphenhydramine, biperiden, or trihexyphenidyl, as well as benzodiazepines, can lead to an improvement in EPS. Clinicians should be aware of the potential risk of EPS associated with antipsychotic treatment and closely monitor patients for these side effects. Treatment adjustments may be necessary to minimize the occurrence of EPS and improve patient outcomes.

## Data Availability

The data that support the findings of this study are available on request from the corresponding author. The data are not publicly available due to privacy or ethical restrictions.

## References

[1] Tien Y., Huang H. P., Liao D. L., Huang S. C. (2022). Dose-response analysis of aripiprazole in patients with schizophrenia in Taiwan. *Therapeutic Advances in Psychopharmacology*.

[2] Stahl S. M. (2021). *Stahl’s Essential Psychopharmacology: Neuroscientific Basis and Practical Applications*.

[3] Seifert S. A., Schwartz M. D., Thomas J. D. (2005). Aripiprazole (Abilify™) overdose in a child. *Clinical Toxicology*.

[4] Kashihara K., Maeda T., Yoshida K. (2022). Safety and tolerability of aripiprazole in patients with psychosis associated with parkinson’s disease—results of a multicenter open trial. *Neuropsychopharmacology Reports*.

[5] Scheil J., Power D. (2023). What is the risk of extrapyramidal symptoms in patients taking aripiprazole?. *Evidence-Based Practice*.

[6] LoVecchio F., Watts D., Winchell J. (2005). One-year experience with aripiprazole exposures. *The American Journal of Emergency Medicine*.

[7] O Nwokike M., Ghasi S. I., Ogbonna A. O., Ezenwaeze M. N., Ezinwa A. C. (2022). Extrapyramidal symptoms and novel antipsychotic drugs. *International Neuropsychiatric Disease Journal*.

[8] Kumra S., Oberstar J. V., Sikich L. (2007). Efficacy and tolerability of second-generation antipsychotics in children and adolescents with schizophrenia. *Schizophrenia Bulletin*.

[9] Ben Amor L. (2012). Antipsychotics in pediatric and adolescent patients: a review of comparative safety data. *Journal of Affective Disorders*.

[10] Bernagie C., Danckaerts M., Wampers M., De Hert M. (2016). Aripiprazole and acute extrapyramidal symptoms in children and adolescents: a meta-analysis. *CNS Drugs*.

[11] Davies M. A., Sheffler D. J., Roth B. L. (2004). Aripiprazole: a novel atypical antipsychotic drug with a uniquely robust pharmacology. *CNS Drug Reviews*.

[12] Madhusoodanan S., Alexeenko L., Sanders R., Brenner R. (2010). Extrapyramidal symptoms associated with antidepressants—a review of the literature and an analysis of spontaneous reports. *Annals of Clinical Psychiatry: Official Journal of the American Academy of Clinical Psychiatrists*.

[13] O’Neill J. R., Stephenson C. (2022). Antipsychotic-induced laryngeal dystonia. *Psycho Pharmacology Bulletin*.

[14] Kimble M. K., Young L. Y., Kradjan W. A. (2009). Applied therapeutics: the clinical use of drugs. Betsy A et al. *Diabetes mellitus*.

[15] Tintinalli J. E., Stapczynski J. S., Ma O. J., Yealy D. M., Meckler G. D., Cline D. M. (2016). *Tintinalli’s Emergency Medicine: A Comprehensive Study Guide, 8e*.

[16] Lavonas E. J. (2021). *First Generation (Typical) Antipsychotic Medication Poisoning*.

[17] Mazer-Amirshahi M., Porter R., Dewey K. (2019). Prolonged QRS widening after aripiprazole overdose. *Pediatric Emergency Care*.

[18] Carstairs S. D., Williams S. R. (2005). Overdose of aripiprazole, a new type of antipsychotic. *Journal of Emergency Medicine*.

[19] Warstadt N. M., Mohan S., Furlano E. R., Shenker J. H., Gibbs E. P., Smith S. W. (2022). Abilifright: A case report of massive aripiprazole overdose in a toddler. *Clinical Practice and Cases in Emergency Medicine*.

[20] Gupta D., O’Hara C. (2019). Aripiprazole toxicity with a biphasic course of somnolence. *Pediatric Emergency Care*.

[21] Lofton A. L., Klein-Schwartz W. (2005). Atypical experience: a case series of pediatric aripiprazole exposures. *Clinical Toxicology*.

[22] Christensen A. P., Boegevig S., Christensen M. B., Petersen K. M., Dalhoff K. P., Petersen T. S. (2018). Overdoses with aripiprazole: signs, symptoms and outcome in 239 exposures reported to the danish poison information centre. *Basic and Clinical Pharmacology and Toxicology*.

